# Estimation of Eulerian strain from tagged CMR images using band-pass optical flow and HARP

**DOI:** 10.1186/1532-429X-17-S1-P62

**Published:** 2015-02-03

**Authors:** Azza S Hassanein, Ayman M Khalifa, El-Sayed Ibrahim

**Affiliations:** 1 University of Michigan, Ann Arbor, MI, USA; 2 Helwan University, Cairo, Egypt

## Background

Tagged MRI images are usually used for measuring regional measures of myocardial contractility, e.g. strain. Different methods area available for analyzing the tagged images, including harmonic phase (HARP) and optical flow (OF). HARP analysis is widely spread due to its robustness and fast processing, although it fails to measure strain on the endocardial and epicardial borders [1]. The purpose of this study is to investigate the capability of OF [2] for measuring Eulerian strain and compare the results to those from HARP on numerical phantom and in vivo tagged images.

## Methods

**Figure 1:**
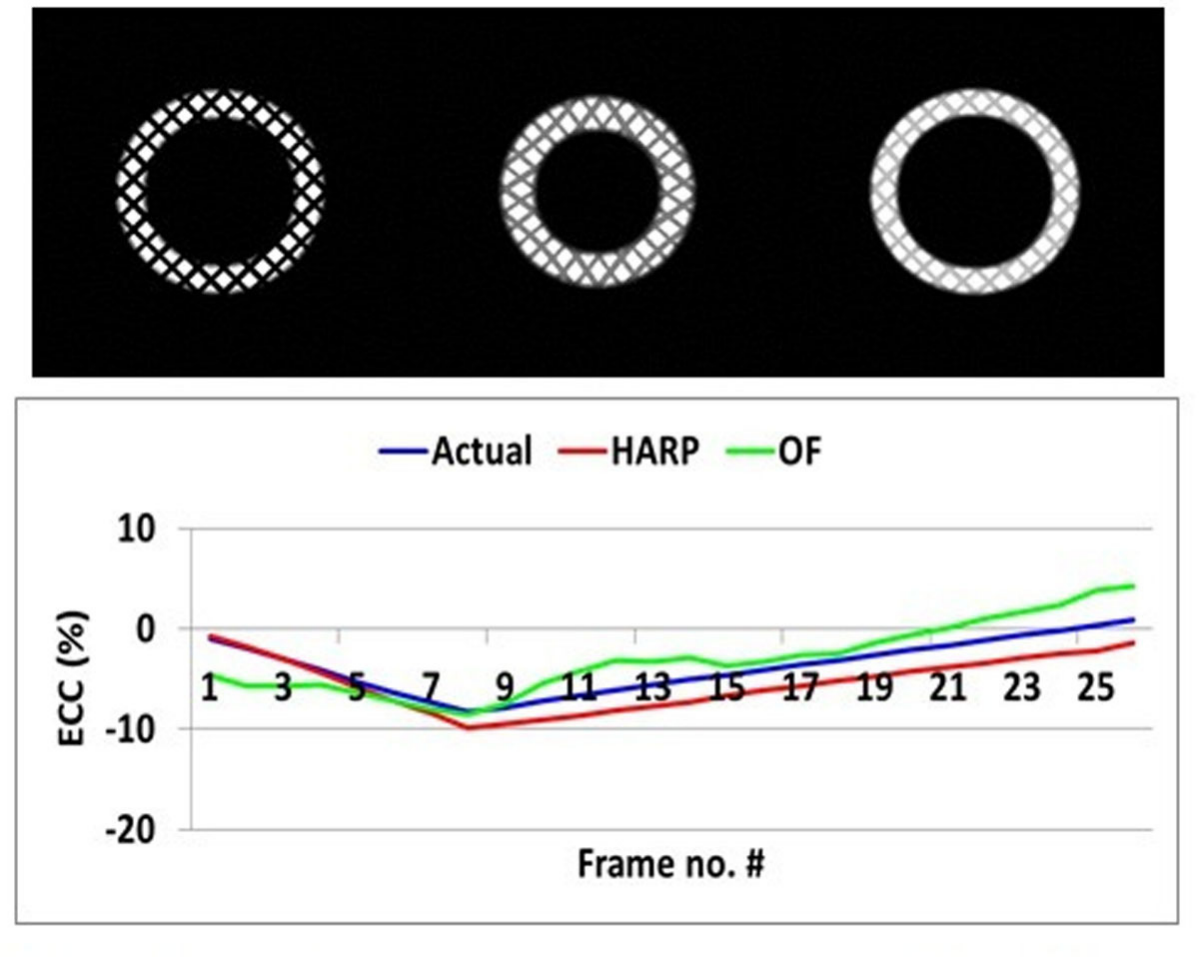
(a) Numerical phantom, showing three frames immediately after the R-wave (left), mid-systole (middle), and late diastole (right). Note the changes in tag deformation and grid relaxation across the cardiac cycle. (b) Circumferential strain measurements in the inferolateral myocardium segment in the numerical phantom from HARP (red), OF (green), and actual phantom (blue).

A numerical phantom was created (Figure 1(a)), consisting of a series of 25 short-axis grid-tagged images of a mid-ventricular slice. The phantom was designed to mimick cyclic myocardial deformation and tagging relaxation during the cardiac cycle. White Gaussian noise was added to the images for realistic simulation. Eleven human subjects were imaged on a 3T scanner to acquire datasets of short-axis grid-tagged images.

Both HARP [1] and band-pass optical-flow (BPOF) [2] techniques were applied on the phantom and in vivo images to measure Eulerian strain. HARP analysis is based on tracking the harmonic phase of the material points in the generated phase images. On the other hand, BPOF estimates velocity by representing the tagged images as 2D sinusoidal brightness pattern multiplying the underlying anatomical brightness field. Thus, in the frequency space, the tagged images consist of various sub-bands located at frequencies related to the product of the implemented sinusoidal frequencies. BPOF calculates velocity from all the sub-bands to provide a robust estimation of strain without the need for mathematical regularization or iterations, which results in spatially homogeneous conditioning of the system of equations, and more accurate results.

## Results

**Figure 2:**
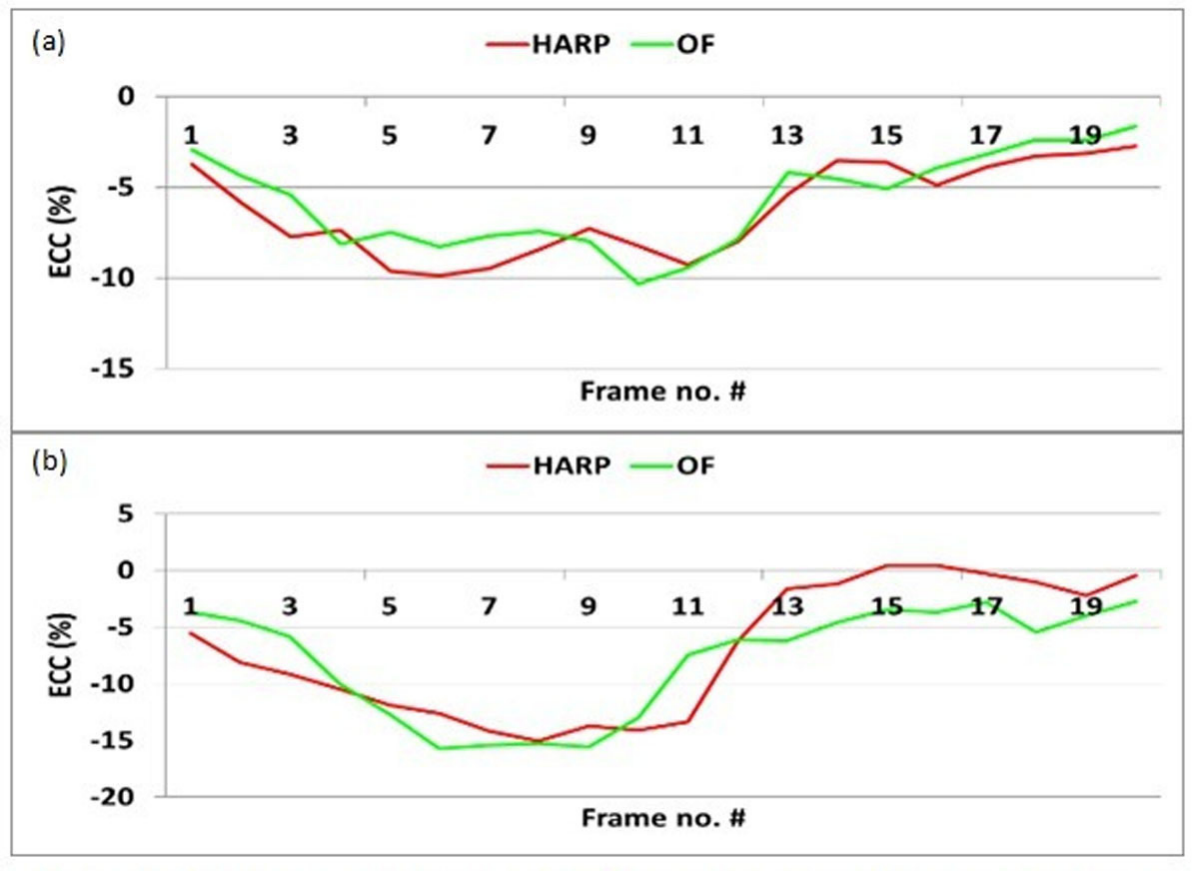
Circumferential strain (Ecc) measurements in human subjects at (a) anteroseptal and (b) inferoseptal myocardial segments using HARP (red) and OF (green).

Figure 1(b) shows circumferential strain measurements in the numerical phantom using HARP and BPOF versus the ground truth grid tags motion. Figure 2 shows the strain results in the human subjects. The results show close agreement between BPOF and HARP without significant increase in processing time.

## Conclusions

BPOF provides reliable Eulerian strain measurements from the tagged images based on ground-truth motion in numerical phantoms and results from human subjects. The BPOF results showed good agreement with the widely used HARP technique without having the HARP limitations, e.g. motion tracking errors at tissue boundaries.

## Funding

N/A.
